# Hospital Annual Meeting

**Published:** 1919-03-15

**Authors:** 


### Hospital Annual Meeting.

### Royal London Ophthalmic Hospital.

At the annual Court of Governors, held last week under the presidency of Mr. H. P. Sturgis, it was reported that the number of in-patients during 1918 was 2,339, including 315 soldiers, for whom 30 beds were set aside. New out-patients last year numbered 36,101, and their attendances totalled 98,186. The daily average number of beds occupied was 113.54, and the average number of days' residence of each patient was 17.72. By the death of Mr. C. D. Marshall while on active service at Bombay the institution has lost one of its most skilful surgeons. Mr. A. H. Payan Dawnay, chief clinical assistant, died towards the end of 1918 after twenty-one years' service at the hospital. Income last year amounted to £19,784, falling short of the expenditure by £250. The most urgent need of the hospital, states the committee of management, is the extension of the accommodation for nurses. Two propositions have been made with this end in view. One is to lift the roof of that part of the hospital building wherein the nurses are at present housed and add another floor. The other is to erect a nurses' home.

